# Developing a clinical pathway to identify and manage cognitive problems in Multiple Sclerosis: Qualitative findings from patients, family members, charity volunteers, clinicians and healthcare commissioners

**DOI:** 10.1016/j.msard.2020.102563

**Published:** 2021-04

**Authors:** Laura Smith, Hannah Elwick, Jacqueline R. Mhizha-Murira, Gogem Topcu, Clare Bale, Nikos Evangelou, Stephen Timmons, Paul Leighton, Roshan das Nair, NEuRoMS collective

**Affiliations:** a School of Medicine, University of Nottingham, Nottingham, NG7 2TU, United Kingdom; b Business School, University of Nottingham, Nottingham, NG8 1BB, United Kingdom; c Institute of Mental Health, Nottinghamshire Healthcare NHS Foundation Trust, Nottingham, NG7 2TU, United Kingdom

**Keywords:** Multiple sclerosis, Stakeholder perspective, Cognitive screening, Cognitive management, Pathway, Qualitative

## Abstract

**Background:**

Cognitive problems are a common and debilitating symptom of multiple sclerosis (MS). Screening and treatment for cognitive problems are recommended, however these are not routinely delivered in UK clinics.

We collected and synthesised stakeholder perspectives to develop a care pathway for cognitive problems in MS and produce a logic model, illustrating how this pathway might operate.

**Methods:**

Forty-nine stakeholders, including people with MS and care providers, participated in semi-structured interviews and focus groups. Participants viewed information that illustrated how the pathway might work and provided feedback. Data, transcribed verbatim and analysed using Framework Analysis, were mapped onto a preliminary logic model and accompanying thematic framework.

**Results:**

The proposed pathway was perceived as helpful in providing standardised support for a neglected MS symptom. Training packages, online cognitive screening, and triaging decisions were viewed as crucial activities. Shared responsibility, a person-centred approach, and addressing the complexity of cognitive problems were important engagement mechanisms. Allocating time during clinic appointments and within staff workloads were essential resources for implementation.

**Conclusion:**

Our co-constructed MS cognitive screening and management pathway will be evaluated for clinical and cost-effectiveness in a trial. However, in the interim, clinicians can adapt and implement this pathway in their own services and evaluate it locally.

## Highlights

• No established care pathway exists for screening and managing cognitive problems.
• Based on stakeholder interviews, we developed a logic model for the pathway.
• The logic model illustrates how a new clinical care pathway could work.
• To work, the pathway relies on shared responsibility and a person-centred approach.

## 1 Introduction

Cognitive problems affect up to 70% of people with MS (pwMS) [1] and can negatively impact quality of life and vocational activities [2], [3], [4]. Consequently, routine screening and management for cognitive problems in MS has been internationally recommended [6], with addressing cognitive problems a ‘top 10′ research priority for pwMS [7].

Despite these calls to action, UK MS services do not have an established care pathway which integrates these recommendations. A UK-wide survey of clinicians found variation in cognitive assessments used, often with inappropriate screening tools used rather than the recommended tests [8]. We also found a lack of consistency in reporting cognitive rehabilitation interventions, particularly regarding the content of interventions and their underlying framework [9]. These issues make it difficult for healthcare services to consistently and systematically implement cognitive screening and rehabilitation [10]. To address this gap, we aimed to develop a multi-agency, co-constructed, clinical pathway to forge a consensus on screening and managing cognitive problems in MS, and how to go about this.

**Fig. 1:**
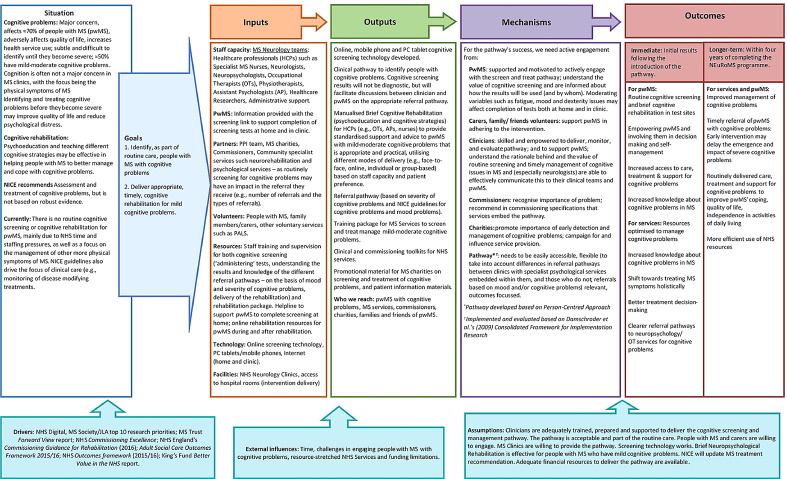
Initial Logic Model.

Medical Research Council (MRC) guidelines stress the importance of theory in developing and evaluating complex interventions [11]. Here we propose an initial logic model (Fig. 1) which depicts the theory underpinning a screening and management pathway for cognitive problems in MS. Our model was informed by literature reviews [12], [13], [14], theory (e.g., Behaviour Change Wheel [15]), Patient and Public Involvement (PPI), clinical experience, and service realities. It adopts a **Situation**-**Inputs**-**Outputs**-**Mechanism**-**Outcome** configuration [16].

**Situation** describes the contextual features that pre-date the introduction of the pathway; including the high prevalence of cognitive problems, lack of standardised screening and support for cognitive problems (particularly mild-moderate problems [17,18], and healthcare recommendations which drive the focus of clinical care (e.g., NICE recommends screening and managing cognitive problems, but this is not based on robust evidence and does not refer to a particular assessment or treatment [19]). **Inputs** are the resources required to support the pathway. **Outputs** describe the products created by the activities of the pathway. **Mechanisms** are mediating factors and obstacles between the introduction of the pathway and the resulting outcomes (e.g., stakeholders need to be engaged and supported; and the pathway accessible and flexible for implementation). **Outcomes** describe what results from the pathway. The specifics of these factors can be found in Fig. 1.

Logic models are dynamic, they represent working hypotheses and are modified iteratively as new insights emerge based on primary (e.g., stakeholder consultation, evaluation studies) or secondary data (e.g., existing literature/policy) [20]. Conducting primary research with key stakeholders can enhance theoretical understanding of the processes of change [21]. Exploring the views of key stakeholders at an early stage also helps to produce a co-constructed output [22] that empowers and engages stakeholders [23].

Here, stakeholder perspectives were used to develop a multi-agency cognitive screening and management pathway. This study is part of the Neuropsychological Evaluation and Rehabilitation in Multiple Sclerosis (NEuRoMS) project [24] which will evaluate the efficacy of this pathway across six UK MS clinics.

## 2 Material and methods

Ethical approval was granted by the University of Nottingham Faculty of Medicine and Health Sciences Ethics Committee (reference: 263–1903). All participants provided informed consent prior to data collection.

### 2.1 Recruitment

We sought to interview a broad range of stakeholders with complementary perspectives on a screening and management pathway for cognitive problems, we selected purposively from those willing to be interviewed to generate this.

Eligible participants were 18 years or older, able to communicate in English and provide informed consent. Our stakeholders included pwMS (diagnosed with MS), family members (relatives or carers for pwMS), MS charity volunteers, clinicians (neurologists, MS nurse specialists, neuropsychologists, occupational therapists (OTs) and neuro-physiotherapists working clinically with pwMS), and healthcare commissioners (working within a Clinical Commissioning Group that commission National Health Service [NHS] services).

PwMS, family members and charity volunteers were recruited through PPI networks, social media, MS charities, and by word of mouth. Clinicians and commissioners were recruited through professional networks.

Participants were contacted by email or phone and invited to a focus group or an interview (in-person, or via telephone/video conferencing), based on their preference.

### 2.2 Data collection

Semi-structured interviews were conducted by authors JMM, RdN, NE and HE (see Supplementary Materials for interview schedules). The focus group with pwMS was co-facilitated by our PPI partner CB, to enhance data richness [25]. Interviews were audio-recorded and transcribed verbatim.

Participants were shown several resources to illustrate how the pathway might work and were asked to provide feedback. These included brief video demonstrations of digital cognitive screening tests (Paced Auditory Serial Addition Task [PASAT] [26], Symbol Digit Modality Test [27], Stroop [28]); examples of face-to-face assessments (Word List Learning [29], Verbal Fluency Test [30,31], Trail Making Test [32]); and, a self-report questionnaire (Multiple Sclerosis Neuropsychological Questionnaire [33]). The initial logic model (Fig. 1) was also shared.

### 2.3 Analysis

Anonymised transcripts were analysed on NVivo 12 using the Framework approach [34]. The logic model informed a working analytical framework (see Fig. 1 and Supplementary Materials for coding scheme) and data were mapped onto this. Review of the mapped and organised data informed a revision of the logic model based on the findings below.

Yardley's evaluative characteristics for good qualitative research were applied [35]. Regular team discussions were also held to modify the coding scheme to better represent the data [36]. Summary data were presented to a PPI group to sense-check our interpretations.

## 3 Results

### 3.1 Study participants

Forty-four participants were interviewed (25–75 min), and 5 pwMS participated in the focus group (125 min). Characteristics for non-clinician stakeholders are presented in Table 1. Participating clinicians included four neurologists, MS nurse specialists, OTs and neuropsychologists each, three healthcare commissioners, and one neuro-physiotherapist. Recruitment stopped when we fulfilled our purposive sampling criteria and established data from a range of different stakeholders.

**Table 1:** Participant characteristics for non-clinician stakeholders.

	People with MS- Interviews (*n* = 15)	People with MS- Focus group (*n* = 5)	Family Members (*n* = 5)	Charity Volunteer (*n* = 4)
Age *M*(±)	48(9.5)	49.6(7.1)	54.2(19.9)	42.3(7.6)
Gender Female	12(80%)	4(80%)	1(20%)	3(75%)
Ethnicity White	14(93%)	5(100%)	4(100%)	3(75%)
*Education level* GCSE A Level Degree Higher degree Other	3 2 4 4 2	2 0 0 2 1	0 1 3 1 0	0 0 3 1 0
*Employment* Full-time Part-time Not employed Retired Voluntary full/part-time Full-time education	4 3 4 4 0 0	2 3 0 0 0 0	1 1 0 1 1 1	0 2 0 0 4* 0
Time since diagnosis *M*(±)	12.9(10.3)	12(8.7)		
*MS sub-type* Relapsing-remitting Primary progressive Secondary progressive Unknown	9 1 4 1	4 0 1 0		

Note: * 2 of these participants work part-time and volunteer part-time.

### 3.2 Overview

There was consensus on the current situation - cognitive problems were reported as prevalent and disruptive, and stakeholders recognised the need to address these problems using a standardised pathway. Discussions therefore focused on how the pathway would work.

Data were organised into **overarching themes** (pre-defined logic model configurations), ***themes*** (core patterns) and *sub-themes* (further depth). Key results are presented for each overarching theme (**inputs, outputs, mechanisms, outcomes**) and supporting data are displayed in Table 2, Table 3, Table 4, Table 5, with superscript numbers linking relevant quotes to the text. When multiple participant groups endorsed a sub-theme the term ‘stakeholders’ is used, otherwise the relevant group of stakeholders is specified (e.g., pwMS).

### 3.3 Inputs (Resources)

***Clinical Staff*** were recognised as an important resource. Stakeholders pointed to *‘competition’ between symptoms during clinic appointments*, with limited time to address multiple MS symptoms^1^. Clinicians commented on the need to monitor drug treatments during the appointment, which took precedence over dealing with cognitive problems^2^. Physical symptoms including spasticity, bladder and bowel issues were often prioritised over cognitive problems^3^.

*Pressured workloads* were also raised, particularly those of MS nurses^4^. Stakeholders reflected that any new pathway would impact on staff time^5^ and that it is unclear who might have the capacity to deliver this^6^.

***People with MS*** thought they would need to expend *mental and physical resources* to engage in the pathway. Travelling to hospital requires time, effort and planning^7^. Digital technologies are also needed to access cognitive screening^8^. Further time commitments may be necessary from those attending multiple rehabilitation sessions^9^. ***Clinic facilities***, including *technology* to host the cognitive screening (e.g., computer tablet, WiFi access^10^), and a *clinic room* where screening and support sessions could be completed^11^, was also identified as a key resource.

Clinicians and commissioners thought *understanding* existing ***costing and commissioning*** frameworks and having a *strategy* in place to show how the proposed pathway addresses these drivers^12^ would help ensure the pathway is adequately resourced.

**Table 2:** Key **Input (Resource)** themes and sub-themes with supporting data.

*Theme*	Sub-theme (Context)	Sample of coded text
***Clinical staff***	*Clinic appointment* Time allocated to cognitive symptoms during limited clinic appointment	1. And in our symptom management clinics where they have half an hour appointments, in that appointment we will be looking at bladder, bowels, fatigue, mobility, spasticity – you name it, it's addressed in that. And if you touch on cognition, but again we don't have time to sit there and go through a proforma or anything like that or any kind of referral. MS Nurse MS07
		2. There's these new medicine, I give you this medicine, has it got any side effects, let me tell you about the side effects and so on. So, the competition for time is, do I – at the moment because we didn't have the evidence base for the effective rehabilitation, there seem to be some pressure and probably it will take second priority in people's minds. Neurologist N01
		3. So I usually really just tell her conversation about medication and that's all there's, you know, we have a bit of a tete a tete about that! [Laughs] And that's all there's time for! Charity Volunteer CV05

	*Pressured Workloads* Capacity of multidisciplinary team members	4. Personally my experience of MS…yeah, I don't know how they would have the bandwidth to do this, it would be great if they do, but you know. Charity Volunteer CV05
		5. I would just worry a little bit about timing, if you're doing it with everybody, because if they've got the – they're also very limited on time and they'll also be doing other OT roles as well. Neuropsychologist PS05
6. I think probably the Occupational Therapists are more used to delivering cognitive screening, giving advice on cognition and especially in the sense of how we're using the, you know, mechanisms – so the strategy that you're suggesting – incorporating with everyday life. …I have the impression that they have possibly a bit more time than the MS nurses. Neurologist N01	

***People with MS***	*Physical & Mental* Requires concentration	7. Because it's hard work when you have to get to a hospital appointment, even for those of us that drive and don't have too much physical problems at any one time. It is hard work. Focus Group Male M2 Planning. Focus Group Male M1 Yeah. To get to [hospital 1], plus the expense, it's a big thing. Focus Group Female F1

	*Physical* Requires travel, time away from work, access to technology, mobility	8. They may not have the money to buy the machinery, machines to access the link, they may not be IT literate and they may just be plain right poorly, you know, too poorly to do it. PwMS P02
9. And also trying to get the timing right as well because obviously a lot of people with MS are working so doing – if it's one off group it's easier, but if it's a group over a number of sessions, doing it during working hours is difficult for people to commit to. Neuropsychologist PS03	

***Clinic facilities***	*Technology* Availability of Wi-Fi, tablet, etc.	10. We have a Wi-Fi. We have also NHS Wi-Fi which is free. MS Nurse MS05

*Clinic rooms* Availability and management	11. There is general mismanagement of rooms in the hospital. So once the MS nurses, they always find this space, because they find that the empty room and let's go there for the next half an hour. Neurologist N01

***Costing and commissioning***	*Commissioning frameworks* Understanding, awareness and a strategy to address	12. So what you'd need to do is cost out this programme in each of those areas, work out what their commissioning structures are for each of those areas and be very clear about what the key metrics are in terms of patient outcomes, experience, safety, funding. Commissioner CM03

*Note*: Alpha-numerical codes represent participant ID numbers.

### 3.4 Outputs (Activities)

Stakeholders agreed that ***training*** was important to ensure that staff feel supported and have the skills to deliver the pathway. Clinicians thought training should address *screening* (administering and understanding the cognitive tasks)^1^, *triaging* (interpretation), and *managing cognitive problems* (goal setting, rehabilitation philosophy)^2^. Clinicians raised *supervision* and ongoing monitoring^3^, recognising that post-training support^4^ would be beneficial.

The online ***screening tool*** was a key focus for discussion (see Supplementary Materials – Coding Scheme). Stakeholders felt that screening should be *administered* ahead of a clinic appointment, with the results enabling discussion during the appointment^5^. Most pwMS felt able to access the screening tool online at home^6^ and advocated sending a weblink to access the tool on a digital device^7^.

PwMS understood the relevance of the *cognitive measures* presented to them and thought these were appropriately challenging^8^. However, some found the mental arithmetic task (PASAT) unpleasant^9^. Everyone agreed that cognitive screening should be brief. Clinicians acknowledged the need to balance the sensitivity and brevity of screening tasks^10^.

Clinicians thought that ***screening results*** should be *digitised* within patients’ medical records^11^. They felt that the *feedback report* should include cut-offs to help identify individuals who may require support^12^ and enable discussions around the type and severity of the cognitive problem^13^. Most stakeholders thought the results should be *communicated* face-to-face, at a routine clinical appointment, rather than over the telephone or via letter/email^14^. Communications should be initiated by a neurologist where problems are severe^15^.

Stakeholders recognised the complexity of ***triage*** decisions and referrals. Where present, *concurrent symptoms* such as low mood and fatigue needed to be interpreted in relation to cognitive problems^16^. Stakeholders also highlighted the importance of the *perspective of pwMS*. Triaging should consider how the person was feeling during cognitive screening and any extenuating circumstances (e.g., relapse, technology problems^17^). Stakeholders thought that pwMS should engage in these discussions to reflect upon the *functional impact* of cognitive problems^18^.

Data relating to the ***cognitive rehabilitation/management programme*** reiterated that concurrent symptoms need to be addressed by the pathway, through the provision of relevant information^19^. Stakeholders felt the *content* should include compensatory strategies that can be implemented at home^20^ and gave examples of strategies they thought might work well (e.g., digital technologies^21^) or be less effective (e.g., abstract visualisation^22^).

**Table 3:** Key **Output (Activity)** themes and sub-themes with supporting data.

*Theme*	*Sub-theme* (Context)	Sample of coded text
***Training packages***	*Screening & Triaging* How to support pwMS to complete screening and interpret the results	1. I'm not sure our OTs, even our specialist rehab OTs would have used the digit symbol and some of these, so it's kind of, you know, are introducing something new to people that then will need to interpret that, but I don't think with training, I think that's feasible isn't it. Neuropsychologist PS05

	*Cognitive management programme* How to deliver and set goals	2. Not all nurses have been through rehabilitation unit or rehabilitation training to kind of be aware of the notion of goalsetting and monitoring and motivational – you know. Neurologist N03

*Supervision* Ongoing monitoring and support	3. So if this is just an MS nurse working on their own then they might find it harder to make some of those decisions unless they've had some solid training and some supervision, ongoing supervision just to, you know, flush it out a bit. Neuropsychologist PS05
	4. I think it does definitely need to have a review and monitoring built into that. Neuropsychologist PS03

***Screening tool***	*Administration* How to enable completion of screening ahead of routine appointment	5. That would be an ideal, digitalising the assessment pre-appointment. That would be wonderful…so when they come into clinic we have got everything there. MS Nurse MS05
		6. Interviewer So you wouldn't have any problems filling that in and accessing the link. Carer C20 No, no, not at all, I'd be alright.
		7. Which is obviously brilliant because the whole thing about a paper link is that someone then has to type it in. And as much as possible, isn't it, you want someone to have the link on their device so they just have to click on it. Neurologist N03

	*Cognitive measures* Include short, sharp assessments that avoid mathematics	8. Interviewer Did those tests seem relevant? PwMS P20 Yeah, very much so, yeah. Especially the one with the colours and the - Interviewer The Stroop test. PwMS P20 Yeah, you know, it's quite profound how your brain works because, you know, somebody without MS you still kind of have to really think one thing but you're having to override it. I think that's a really good test
		9. Because I've got to think of too many numbers so I can't focus on the number that I need to focus on because I've just done the total. So I will have forgotten that number. I just couldn't do that. Focus Group Female F1
10. Your balances a bit, isn't it, it's finding something which is quick but MS nurses will be able to do with everybody quickly as part of their clinical interview while still being meaningful enough. Neuropsychologist PS03	

***Screening results***	*Transfer* Electronic transfer to clinical team	11. So probably for us it would be emailing to the MS coordinator, who would upload it onto the patient's EPR so that it was there as an electronic document and then it would remain so, as opposed to lost in someone's email and never available again. Neurologist N03

	*Feedback report* What should this include	12. I think if we could categorise them really in a binary way or in these three categories, I think that would be excellent. That's very practical and I think we all know pretty well who we're thinking of when we're thinking of these categories. Neurologist N02
		13. I mean, it might be useful to know in roughly what sort of domain we're talking about the deficit as being, you know, so whether it's a memory problem or whether it's – I don't know – been a processing problem or maybe something roughly categorising it a little bit further rather than just severity. Neurologist N04

*Communication* How to communicate screening results to pwMS	14. I think face to face is probably better than an email. Carer C20
	15. Well it depends what the result is. If it's very severe, you're going to need a consultant I think. Focus Group Male M2

***Triage***	*Concurrent symptoms* Interpret cognitive performance considering concurrent symptoms	16. You will need somebody who's quite skilled at interpreting the different components you've discussed, so the mood and the cognitive, to think about how best to manage their difficulties. Neuropsychologist PS03

	*Perspective of person with MS* Allow pwMS to indicate how they are feeling	17. Everything's all right, or you might be having a bad day but you picked up this. How do you feel about how this goes for you every day? Is this actually a problem or was it just, you know, that answer on the test? PwMS P27

*Functional impact* How do cognitive problems affect pwMS	18. I think as long as it's then not prescriptive and it's not taken at that value of it being the person in front of them is sitting there saying ‘I'm struggling at work’ and we turn round and go ‘but the screens don't show us anything’ and then that's taken as part of I suppose a triangulated discussion which you'd hope any clinical team would facilitate. Neuropsychologist PS05

***Cognitive management progr*****a*****mme***	*Content* What should the programme cover	19. And it could also provide a bit of information, you mentioned fatigue earlier and that having an impact sometimes. Charity Volunteer CV01
		20. Skills, different techniques, to help us and then you perhaps try and test it. Focus Group Male M2
		21. The way that people are making decisions, and in the way that they're managing their condition or not managing their condition effectively. Occupational Therapist OT02
		22. If it's abstract it's very difficult for them to take, say reading something, or being given a handout to applying it. Neuropsychologist PS03

*Note*: Alpha-numerical codes represent participant ID numbers.

### 3.5 Mechanisms

The ***complexity of cognitive problems*** was raised as an important mediator. Stakeholders highlighted the *interdependence of symptoms* and recognised that stress and fatigue can exacerbate cognitive problems^1^. Similarly, cognitively demanding activities left pwMS feeling fatigued and drained^2^. Stakeholders thought the pathway should recognise that some cognitive problems will stem from brain damage *driven* by MS, whilst others are a secondary reaction to living with MS^3^. Clinicians thought this was an important distinction to observe^4^. Stakeholders suggested the pathway should also acknowledge that *individual differences* could influence cognitive performance and the effectiveness of support programs, and the perspective of related informants need to be addressed^5,6^.

***Engaging pwMS*** addresses their reactions towards the pathway. Stakeholders felt pwMS should be informed about the *rationale for the pathway*. Explanations should reassure them that the pathway is meaningful^7^ and clarify how it will inform their clinical care^8^. Stakeholders thought information should be *clearly communicated* avoiding medical jargon^9^.

The *timing* of the pathway is an important mediator for pwMS. Stakeholders thought invitations to complete the screening should be aligned with a routine appointment where the results can be communicated without delays^10^. Most pwMS supported the idea of being told about the screening tool in advance of their appointment^11^ but acknowledged some might worry about this^12^. Some thought receiving information about the pathway might overwhelm newly diagnosed patients^13^, while others felt cognitive problems should form part of these early conversations^14^.

Stakeholders indicated that *home-based approaches* (e.g., online screening, telephone follow-ups for the cognitive management programme) would be convenient and less stressful for most pwMS^15^, particularly those in employment who cannot attend multiple appointments^16^. However, home-based approaches were not perceived as feasible for all^17^; stakeholders thought *access to additional support* (telephone or face-to-face in-clinic) would promote engagement^18^.

***Engaging clinical staff*** encompassed clinicians’ reflections on which team members they thought *responsible* for addressing cognitive problems. Some thought MS nurses and neurologists would not consider cognitive problems as part of their role^19^ or see this as beyond their expertise^20^. Other clinicians perceived cognitive problems as a shared responsibility and thought the pathway should advocate a team-based approach^21,22^. Clinicians acknowledged that staff have different priorities and thought *willingness to prioritise cognitive problems* would promote acceptance of the pathway^23^.

Stakeholders thought the pathway should ***foster shared values***, including a *person-centred approach* where care is tailored to the needs of the individual^24^. The pathway should recognise the differing impact that cognitive problems have^25^ and ensure support strategies are meaningful to the individual^26^. Some stakeholders recommended adopting a holistic approach to care^27^, incorporating physical and psychological wellbeing^28^.

The pathway should empower pwMS to be *proactive* and to take actions which target milder cognitive problems^29,30^. However, stakeholders also acknowledged some pwMS will not be motivated to engage with this because of fatigue and cognitive problems^31^.

PwMS thought the pathway should promote a *positive* outlook and inspire them^32,33^. However, clinicians recognised the need to *manage expectations,* e.g., manage problems rather than restore abilities^34,35^. These discussions can be frustrating for pwMS who want their cognitive abilities restored^36^. Stakeholders thought appropriate information might temper unrealistic expecations^37^.

**Table 4:** Key **Mechanism** themes and sub-themes with supporting data.

*Theme*	*Sub-theme* (Context)	Sample of coded text
***Complexity of cognitive problems***	*Interdependence of symptoms* e.g., cognition mood, fatigue	1. Her cognitive issues come when she's under stress, so she breaks down into this almost jelly like state. Carer C20
		2. There's lots of tabs open in my head, that's the only analogy I can put it down to and that creates the stress and then it's almost like I can't think straight and my head is like just all over the place and then that completely fatigues me. PwMS P20

	*Nature of cognitive problems* Brain-based or secondary reaction	3. There's also lots of questions about whether it's associated with their mood or whether it's to do with their MS or some other issues too, so those questions often come up quite a lot. Neuropsychologist PS03
		4. Sometimes I think we tend to think about real and not real cognitive problems and give the impression that we think the real ones are the ones related to scanning and your depressed, your tired, your sleepy patients do have cognitive problems, it's just the means to address them are likely to be different. Neurologist N03

*Acknowledge individual differences* Personal circumstances	5. I guess that's my question, like, how do I know I've got cognitive problems over and above the average 40 something year old. Charity Volunteer CV05
	6. We do have people who there can be a temptation sometimes to overegg your cognitive problems. Neurologist N03

***Engaging people with MS***	*Rationale clearly explained* Describe pathway and what it involves	7. Someone might look at that and think “I don't see why I'm doing this. I don't understand why I'm being asked to do this. This has no bearing on my life. I'd never do a test like this in my real life”. PwMS P27
		8. I think that, no, it's just explaining about the benefits they can get from it and that sort of thing. Carer C20

	*Instructions* Clear, concise instructions	9. Yeah, I think definitely simplicity is definitely, yeah, the way to do it, yeah. PwMS P02

	*Timing* Align screening with an upcoming appointment and avoid point of diagnosis	10. If it's that sort of level and it can get fed back fairly quickly at the next appointment, that – ‘cause you don't want to be sitting worrying about it, you want to have something pretty quick. PwMS P27
		11. I think maybe if they'd told me in advance ‘we're going to do a very basic, you know, cognitive test, it's nothing to worry about, we'll explain it, please don't do any preparation, but just be relaxed about it’, I think that probably would have helped. Charity Volunteer CV05
		12. I think if I'd known about in advance, you know, I might have then overthought it, to be honest. Charity Volunteer CV05
		13. I think as people have said before, it's like what stage you're at and whether you want to look or whether you need to look. Focus Group Female F1
		14. I had no idea about the cognitive things until later on and that was a nasty shock. Yeah, I think being more upfront about things might be, would be useful. PwMS P27

	*Home-based completion* Remote screening allows flexibility and convenience	15. I think so. I think a lot of people would be happier doing that at home. It's more of a – yeah – it's that less stressful situation, you know. Carer C20
16. Doing it during working hours is difficult for people to commit to. Neuropsychologist PS03	
17. I can imagine there's people who are really not au fait with computers, with it all becoming paper free and that stuff. PwMS P28	
18. I think discussion in person one to one, whether that be a consultant or MS nurse, is probably the way to start it off. PwMS P27	

***Engaging clinical staff***	*Perceived responsibility* As an individual and within a clinical team	19. I am trained to give medicines. So, why to see patients and make sure that they have all medicine's correctly. So there will be some doctors that will not be keen doing this 2 min in a consultation. Neurologist N01
		20. I think that it's not part of their role, they wouldn't see it as part of their role and it's slightly out of their competency I suppose…it's not traditionally seen as a nurse thing, I don't think, it's more a psychology, an occupational therapist, you know, an OT thing, yeah. Neuropsychologist PS01
		21. I actually do think it needs to be you know, every symptom and management kind of needs to be sort of kind of responsibility for everybody. Occupational Therapist OT04
		22. So it's really useful to have that team approach to cognitive difficulties. Neuropsychologist PS03

*Willingness and motivation* To adopt an alternative approach	23. Yes, we absolutely value the importance of cognition. And that would be no problem at all from our perspective, in terms of making that a key priority. Occupational Therapist OT02

***Foster shared values of the pathway***	*Person-centred* Individualised	24. Not the same thing works for everybody, so I think a kind of basic starting point and then personalise it after that would be a good idea. Charity Volunteer CV01
		25. How much it's impacting somebody's life, so you can have, you know, quite mild difficulties but that really impact on somebody's life, or you can have moderate difficulties which aren't really impacting. Neuropsychologist PS03
		26. I suppose it's meaningful to that person, rather than it just being a lot of suggestions thrown at them, that it is meaningful to that person. Neuropsychologist PS05

	*Holistic* Care for the person as a whole	27. We want to ensure there's parity, that these people get parity of esteem, that they're whole system. Commissioner CM02
		28. So there's always been very holistic, very focused on physical rehab as well as psychological and cognitive. Neuropsychologist PS04

	*Proactive* Initiate and act	29. I feel like that could have quite a protective element to it …and it helps them, it empowers them to speak about their difficulties as well with language that they understand. Neuropsychologist PS05
		30. It's to help and it'll help yourself and it'll help others and just go that way. Carer C20
		31. So if somebody is really, really depressed, again through struggles with their memory and they will probably struggle with initiation as well because they'll be so low, so actually probably implementing some memory strategies might be difficult for them. Neuropsychologist PS03

	*Positivity* Encourage a positive attitude	32. Somebody who's like inspiring, positive, you know, that's what I would love. Focus Group Female F1
		33. It's about retaining a positive mind and a positive mindset. PwMS P20

*Manage Expectations* Coping with cognitive problems - not retraining cognitive skills	34. It's about this is a way of managing and understanding, not a way of getting rid of difficulties. Neuropsychologist PS05
	35. But I think it's very important in terms of the language we're giving MS nurses and other professionals who are then going to be feeding this back and doing cognitive rehabilitation is the idea that coming on these four sessions, it's not going to make this any better, but it may help you to live with it better. Neuropsychologist PS05
	36. I want to recover, yes, I do want to compensate and find other strategies in the meantime, but I still want to feel that it's something I can regain and rebuild. Charity Volunteer CV05
	37. And there are really difficult conversations to have with lots of people we work with and you're going ‘actually there's no evidence for that’. Neuropsychologist PS05

*Note*: Alpha-numerical codes represent participant ID numbers.

### 3.6 Outcomes

We elicited ‘***short-term***’ outcomes of the pathway from the data, including improved access to standardised clinical care for cognitive problems. Clinicians spoke about the lack of *formalised guidelines* for MS services^1^ and thought the pathway would address this^2^. Clinicians and commissioners felt the pathway would facilitate *conversations* about cognitive problems by providing information about patients’ symptoms^3,4^. These *conversations* were also perceived positively by pwMS^5^, who valued the opportunity to discuss cognitive problems^6^ and have their concerns validated^7,8^.

Some stakeholders acknowledged that anticipating the screening results could worry pwMS^9^. Moreover, being *reminded of symptoms* and noticing cognitive decline might be upsetting^10,11^. Consequently, some pwMS may disengage with the pathway^12^.

‘***Longer-term***’, stakeholders thought being able to understand and manage cognitive problems would *improve quality of life* amongst pwMS^13,14^. Earlier detection and management of problems might even *prevent* deterioration^15^ and prolong independence^16^. Stakeholders indicated that early detection and management could promote *efficient use of NHS reso*urces^17,18^. Home-based cognitive screening was also considered efficient, reducing the time required with a clinician^19^. Stakeholders reflected on a *potential increase in referrals to psychological services*^20^, which could overwhelm already pressured services^21^.

**Table 5:** Key **Outcome** themes and sub-themes with supporting data.

Theme	*Sub-theme* (Context)	Sample of coded text
***Short-term: Service***	*Standardised care* Formal guidelines and referral pathways	1. Cognitive problems in people with MS they aren't routinely identified within normal practice, so no standardised way. Neuropsychologist PS03
		2. I think if there's a tool there to use, I think it would facilitate, I can see it leading to more! Neuropsychologist PS05

	*Opportunity to discuss cognitive problems* Confidence and competency to discuss	3. The patient would complete this and it would give more information to whoever is seeing them at their next appointment to further their conversation. Commissioner CM03
4. I think if it creates a conversation, I think anything is helpful and if it prompts the conversation in the direction of triangulating that with the patient experience and any observations that the team or families can offer, then it's doing the right thing. Neuropsychologist PS05	

***Short-term: People with MS***	*Opportunity to discuss cognitive problems* Better able to understand and manage their condition	6. It helps them, it empowers them to speak about their difficulties as well with language that they understand. Neuropsychologist PS05
		7. It feels so subtle that I think when those people mention that, it can be so easily dismissed as it's not impacting that much, but actually it can have a huge impact. Neuropsychologist PS05
		8. I think it is about facing it on, it's not about keeping it down there, you have to face that you have had some changes and run with it, rather than hiding it, so I think questionnaires are good. PwMS P20

	*Remind person of their symptoms* Being reminded of symptoms could be upsetting	9. In terms of doing a test on your own at home feeling like you've really struggled with it and then not having anyone to talk to about that until you're at your appointment, which is then filled with lots of other things, could feel quite isolating. Neuropsychologist PS05
		10. It depends what sort of relationship they've got and obviously their disability as well, you know, how it's impacted so it can be quite emotional for some people I suppose. PwMS P20
		11. And it's basically whether it is actually upsetting to some people to realise just where you were to where you are now, and that really upset me, from that point of view. PwMS P28
12. There are some people who, for a number of reasons, do not want things measured or recorded. Neurologist N03	

***Longer-term***	*Quality of life* Improved confidence and productivity	13. I suspect that if people felt their cognition was better, they would have more confidence and more willingness to go out and do things and try things. PwMS P27
		14. And the idea that if you invest in a strategy to make life a bit easier then you probably may not get fatigued as quickly. Neuropsychologist PS05

	*Prevention* Earlier detection and intervention to reduce impact of cognitive problems	15. It's like why wait for the problem, just, you know, if people knew about that then they could get, they could work on it and get better and slow down the progression of cognitive depletion. PwMS P20
		16. I I If they understand those changes earlier and they can learn the strategies then potentially that might protect employment. Neuropsychologist PS05

	*Efficient use of resources* Optimise NHS resources	17. I mean, is this going to stop people ending up having unnecessary attendances or admissions into hospital, or is it going to just keep people more able to live at home for longer on their own or whatever it is. Commissioner CM03
		18. Yeah – and also the not having to – if they're not going to some services then they don't have to travel and the cost and impact that it has on that. Commissioner CM03
		19. Proposal of having patient to carry out assessment online at home, and is not coming to me, to clinician, I think is an excellent idea. MS Nurse MS05

*Potential increase in referrals to psychological services* Pathway could overwhelm already pressured services	20. This new activity that you are going to be potentially offering here is going to be – this in itself is going to cause more outpatient appointments, isn't it, it's going to cause more – it's going to encourage more appointments, or is this in Primary Care?
	- so there'd be more activity in Secondary Care on the back of the fact that people would be called in for cognitive appointments. Commissioner CM03
	21. But for us actually we have a massive delay to CBT and talking therapies. Neurologist N03

*Note*: Alpha-numerical codes represent participant ID numbers.

## 4 Discussion

**Fig. 2:**
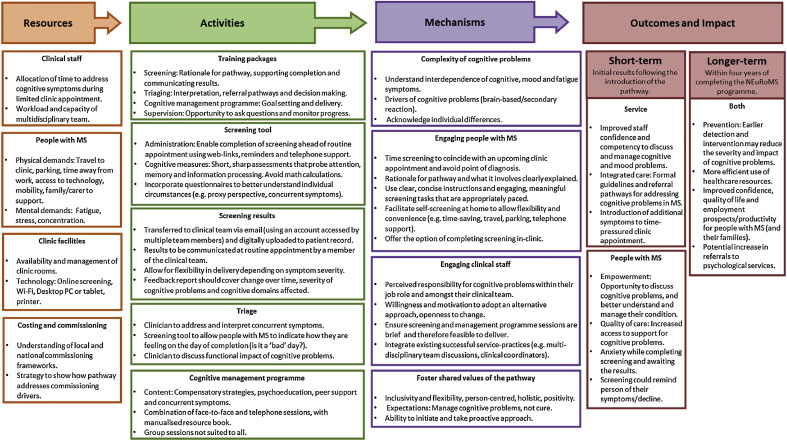
Revised Logic Model Based on Stakeholder Input.

Stakeholder feedback confirmed that staff time, training packages, brief online screening tasks, and person-centred support for cognitive problems are important elements in the pathway. Improved access to care and clearer referral pathways for cognitive problems were still thought of as possible outcomes (as in Fig. 1). However, our data also offers new insights that challenge the initial logic model and enabled us to consider implementation more directly. Fig. 2 reflects a revised logic model that could inform subsequent service development.

‘Resources’ replace ‘Inputs’ to reflect the importance of stakeholder investment. Our revised model recognises the need for staff to allocate time for the pathway during already stretched clinic appointments and pressured workloads. Stakeholders felt the capacity of MS nurses was particularly limited and this led us to reconsider which team members are best placed to support a new pathway. Other staff (e.g., psychologists, OTs) can be involved with appropriate training. Kalb et al. [6] acknowledge insufficient resources and highlight the lack of adequately trained clinicians as barriers for addressing cognitive problems. Training was therefore retained within the revised model with cognitive screening, triaging, management, and supervision packages now specified to reflect our new insights and explain how services can equip multi-disciplinary team members to deliver the pathway. Improved awareness of the importance of addressing cognitive problems within healthcare drivers/ recommendations and accepting shared responsibility for cognitive problems as part of comprehensive care provision amongst the wider neurology community will also help engage clinical staff with the pathway.

The ‘Activities’ component (previously labelled ‘Outputs’) supports recommendations that computerised testing is a means to implement cognitive screening in routine care (13). Electronic data gathered in this way may be integrated within patient records to promote routine monitoring [37]. Our study builds on this by demonstrating how and when cognitive screening could be undertaken (i.e., remotely prior to an upcoming clinic appointment with the option to access additional support). This may facilitate clinical implementation and sustainability.

Our revised logic model reflects the nature of cognitive problems and individual circumstances as important mediating factors. Medications, age, selection/scoring of cognitive assessments, comorbid health conditions and concurrent symptoms can affect cognitive problems [6]; assessing these factors is therefore recommended for optimal cognitive management [38]. Interpreting and contextualising results (because of this) might be challenging and staff training is needed to facilitate consultations and aid triage decisions. Psychoeducation focussing on concurrent symptoms should also be provided to pwMS.

Our findings provided insights into the content of the cognitive rehabilitation programme. This should be person-centred, encompassing psychoeducation and behavioural strategies to help pwMS cope with cognitive problems - not retrain cognitive skills. The rehabilitation programme was therefore relabelled as ‘cognitive management’ to better reflect the content and ethos of the programme. This is consistent with previous research which has demonstrated improved mood and self-reported memory problems, but no restorative cognitive effect following rehabilitation in MS [39].

Stakeholders thought a combination of face-to-face and telephone-based programme sessions would be preferable; Goverover et al. [12] have shown increased adoption associated with such combined approaches, which has since been accelerated and optimised by the COVID-19 pandemic [40]. Online cognitive screening and telephone-based support will be important in engaging stakeholders and in utilising clinic resources efficiently.

Stakeholders thought the pathway would help facilitate conversations about cognitive problems but recognised that implementation could increase referrals to psychological services. Our revised logic model now acknowledges this possibility as a ‘longer-term’ outcome.

Stakeholders also felt the impact of cognitive screening on the wellbeing of pwMS should be considered. Most pwMS valued the opportunity to discuss cognitive problems but acknowledged that some could feel anxious (about screening) and depressed (if the results indicated a problem). These potential outcomes will be monitored as part of NEuRoMS. Ultimately, stakeholders felt referral pathways could help optimise resources (staff can treat those most likely to benefit from their help), prevent mild cognitive problems becoming more severe, and prolong functional independence - all key priorities in managing long-term neurological conditions [41].

In line with MRC recommendations [11], we took a theory-based approach to intervention development to model the causal processes of the pathway. Conducting new primary research with a varied sample of stakeholders offers an in-depth and diverse understanding into how the pathway might work, recognising important mediating factors for implementation. The resulting pathway is now more relevant and theoretically sound, and therefore more likely to be implemented by stakeholders [23].

Nonetheless, our results are based on data gained from a research sample. Other stakeholders might offer further nuance to our understanding - for instance, we did not include IT specialists who would be responsible for data linkage from the screening results to the patient notes and ultimately integrating the pathway within existing IT systems. This, and insight from subsequent pathway piloting, will contribute to the iterative development of the logic model.

Our model is thus a blueprint that healthcare professionals could adapt to suit local circumstances, with local pathways being designed in the nexus of our logic model and local needs/resources. We view the training packages, screening tool, triaging and cognitive management programme (resulting in an integrated care pathway) as core elements to be retained across healthcare systems/pathways. However, availability and commissioning of resources will differ across healthcare models. For those low-income and middle-income countries where patients do not routinely receive disease modifying therapies, this might be prioritised in terms of funding [42]. Access to technology (e.g., tablet and Wi-Fi access for screening in-clinic) may also be limited here. Transferring the screening results to the clinical team will also require contextual strategies to integrate the results within existing record keeping systems (e.g., digital software, paper-based files) and ensure accessibility. Engagement mechanisms of timing screening to coincide with an upcoming appointment and providing the option of in-clinic support will also vary, with healthcare systems showing considerable variation in the frequency of consultations (e.g., quarterly versus annually [43]), which will influence the timing of appointments. Our model offers MS clinics the flexibility to work within the constraints of their systems and integrate existing successful practices.

## 5 Conclusions

Existing MS clinical pathways have not been co-constructed with stakeholders and are not based on robust evidence [19,44]. We have developed a multi-stakeholder, co-constructed, clinical pathway for routine screening and management of cognitive problems in MS. Our stakeholders felt that introducing brief online screening tasks (with options to complete in-clinic or at home) and support for self-managing cognitive problems (including mild problems), would improve quality of life for pwMS and streamline NHS resources through earlier detection and intervention. Clinical staff will need to invest their time and require training, while pwMS should be supported to actively participate in the pathway. As part of the NEuRoMS programme, the clinical and cost-effectiveness of the pathway will be evaluated in clinical trials encompassing intervention fidelity, health economics, and process evaluations.

## Funding information

This report is independent research funded by the National Institute for Health Research (Programme Grants for Applied Research, Neuropsychological Evaluation and Rehabilitation in Multiple Sclerosis – Developing, evaluating and implementing a clinical management pathway (NEuRoMS), RP-PG-0218–20002). The views expressed in this publication are those of the author(s) and not necessarily those of the NHS, the National institute for Health Research or the Department of Health and Social Care.

## Disclosure

The Authors declare that there is no conflict of interest.

## Data Statement

Anonymised data that support the findings of this study are available from the corresponding author upon reasonable request.

To facilitate reproducibility and date reuse, we also share our logic models (Figs. 1 and 2), coding schema (Supplementary Materials) and interview schedules (Supplementary Materials).

## CRediT authorship contribution statement

**Laura Smith:** Formal analysis, Writing - original draft, Methodology, Conceptualization. **Hannah Elwick:** Investigation, Writing - review & editing, Methodology, Formal analysis. **Jacqueline R. Mhizha-Murira:** Investigation, Writing - review & editing, Methodology, Formal analysis. **Gogem Topcu:** Writing - review & editing, Methodology, Conceptualization, Formal analysis. **Clare Bale:** Investigation, Conceptualization. **Nikos Evangelou:** Investigation, Writing - review & editing. **Stephen Timmons:** Writing - review & editing. **Paul Leighton:** Conceptualization, Methodology, Writing - review & editing. **Roshan das Nair:** Conceptualization, Investigation, Supervision, Methodology, Writing - review & editing.

## Appendix Supplementary materials

Image, application 1
