# Supplementary material for: Developing a clinical pathway to identify and manage cognitive problems in Multiple Sclerosis: Qualitative findings from patients, family members, charity volunteers, clinicians and healthcare commissioners
Source: Mult Scler Relat Disord. 2021 Apr;49:None. doi: 10.1016/j.msard.2020.102563 (PMC7533050; doi:10.1016/j.msard.2020.102563)
Supplement: Supplementary file 1 [file mmc1.docx]

**Supplementary Materials**

**Interview Schedules**

## NEuRoMS WP1a Semi-structured stakeholder interview schedule – clinicians and commissioners

Note: As per the methodology, the semi-structured interview is only a guide, and is necessarily an iterative process (i.e., it gets modified slightly after each interview, based on the information that participants provide).

**All participants**

Objectives

Nuanced feedback on the various proposed tests, intervention and pathways.

General order

- Introduction:
- Explain purpose of the interview
- Explain confidentiality and disclosure policy.
- Explain how long the interview is going to take- 30-60min approximately.
- Remind participant that the interview will be audio-taped. Also that the interviewer might take notes.
- Warm up
- Ask general questions as icebreakers and establish rapport.
- Carry out the interview
- From easy, more general to in-depth questions
- Cool off
- Straight-forward questions to relax the interviewee
- Thank them for their participation; goodbye

**General questions**

- The proposed model for the screening and management pathway

We want to find out (from **all** participants):

- What would they like to happen in relation to cognitive screening and management in MS services? Exploring how the pathway could be delivered in an ideal world.
- What they think can happen within current NHS services (practical/logistical constraints). Exploring the potential barriers/facilitators to delivering the proposed pathway.
- Complexity (introduce pathway image for pwMS/carers/volunteers and logic model to clinicians/commissioners)
- We are interested in how the service would work and how the process would look like, including how things are organised and how the screening and management pathway is going to work
- Possible screening tools and completion formats (online, PC, tablet), location (at home, clinics), presented as videos of completion (pen and paper and digital) and as a digital version if they wish to try it out.
- If it is feasible and acceptable to complete cognitive assessments at home, and what access to technology and the internet is available
- The intervention content, format and delivery for NHS contexts
- The commissioning of screening and intervention (clinician/commissioner specific)
- The functionality/appropriateness of proposed tests, pathways and outcomes
- Usual care for people with cognitive problems

**Example questions**

1. Using the preliminary logic model (presented to participants in paper format or on a slide) of the proposed cognitive screening and management pathway, can you make any changes to it, based on how you would like the pathway to work?
2. What do you see are the main barriers and facilitators to the delivery of this pathway? What can be done about the barriers?
3. What are your comments about the specific contents of the intervention? What’s missing? What’s not needed?

**Specific questions**

***Commissioners***

- What aspects of the services would they consider commissioning?
- Who would be best placed to commission and provide such services?
- Usual care related to cognitive assessment and intervention
- Who commissions services in their area?
- What are the sorts of things that are being commissioned?
- What other questions should we ask commissioners?
- What do we need to do as researchers to increase the chances of commissioning?

***Clinicians***

1. Current cognitive screening and management in routine practice

- Ascertain the scope of cognitive screening and management in their current services: to get a sense of how services are like currently and getting a flavour of what is missing
- Is there no cognitive screening/management service currently?
- Or do pwMS get a routine appointment with cognition included within that appointment?

1. Appointment letters

- How are routine appointment letters generated/sent to patients?
- Who is in charge of this? If they are not around, who else is responsible for sending out letters?
- What happens to cancellations, including at what point appointments are cancelled?
- Scenario: appointment letter sent 3 months ahead of the appointment, together with the link for patient to complete cognitive screening test. Assessment completed. Appointment cancelled. New appointment arranged. What does this letter say? New appointment scheduled 6 months after screening completed.
- Patient would need to complete screening again (max 3 months in advance of the appointment, ideally)
- How could we resend the link and/or ask the patient to complete screening test again.
- Results indicate severe cognitive problems. What should we do about that?
  - Unpick how patient should be notified (and by whom) in relation to newly scheduled appointment

1. Proposed cognitive screening and management pathway

- How can we make the cognitive screening and management pathway work in the context of how services are organised at the moment? Purpose is to gain a sense of organisational and professional boundaries
- Finances
- Room booking
- Time (for MS nurses, OTs and neurologists)
- What information should be contained in the cognitive screening results (provide examples)
- We want to ascertain how much information is sufficient – what is ‘too much’ or ‘too little’
- For example cut-offs, summary e.g., no, moderate, severe cognitive problems
- How best to send the cognitive screening results?
- Email?
- Who should receive the results? E.g., MS nurses or neurologists?
- How to transfer the results to patient records
- Access to patient records? If so, what system do they use e.g., system one?
- Clinical content of the intervention: how it should be organised so that it can work?

1. Outcomes

- Outcomes of screening and cognitive rehabilitation programme
- What are the outcomes we should consider? For example: cognition, mood, self-efficacy, quality of life
- Are the following outcomes acceptable?
  - Impact of cognitive problems
  - Cognitive impairment
  - Quality of life
  - Mood
  - Function
  - Self-efficacy
  - Resource-use questionnaire
  - Work-related issues
  - Improved DMT adherence
- What outcomes are we not tapping?

1. Is there a risk that clinicians and/or patients could misuse the screening results?

5.1 if yes, how can those risks be mitigated?

1. Service commissioning

- Who commissions services in their area?
  - What are the sorts of things that are being commissioned?

**Prompts**

Probing questions (Rubin and Rubin, 1995) will be used to transition the conversation from general to specific. That is:

- Requests for extension- e.g. ‘Can you tell me more about....?’, ‘Is there anything else?’, ‘What happened then?’
- Encouraging/prompting questions- e.g. ‘uh huh?’, ‘Yes?’, ‘Please go on’.
- Example questions- e.g. ‘Can you give me an example of?’
- Follow-up questions- e.g. ‘What do you mean by...?’, ‘Would you talk a bit more about...?’

## NEuRoMS WP1a Semi-structured stakeholder interview schedule – people with MS, carers and volunteers

Note: As per the methodology, the semi-structured interview is only a guide, and is necessarily an iterative process (i.e., it gets modified slightly after each interview, based on the information that participants provide).

**All participants**

Objectives

Nuanced feedback on the various proposed tests, intervention and pathways.

General order

- Introduction:
- Explain purpose of the interview
- Explain confidentiality and disclosure policy.
- Explain how long the interview is going to take- 30-60min approximately. Remind participant that the interview will be audio-taped. Also that the interviewer might take notes.
- Warm up
- Ask general questions as icebreakers and establish rapport.
- Carry out the interview
- From easy, more general to in-depth questions
- Cool off
- Straight-forward questions to relax the interviewee
- Thank them for their participation; goodbye

**General questions**

All participants will be asked to provide feedback on:

- The proposed model for the screening and management pathway

We want to find out (from **all** participants):

a. What would they like to happen in relation to cognitive screening and management in MS services? Exploring how the pathway could be delivered in an ideal world.

b. What they think can happen within current NHS services (practical/logistical constraints). Exploring the potential barriers/facilitators to delivering the proposed pathway.

- Complexity (introduce pathway image for pwMS/carers/volunteers and logic model to clinicians/commissioners)
- We are interested in how the service would work and how the process would look like
- Including how things are organised and how the screening and management pathway is going to work
- Possible screening tools and completion formats (online, PC, tablet), location (at home, clinics), presented as videos of completion (pen and paper and digital) and as a digital version if they wish to try it out.
- If it is feasible and acceptable to complete cognitive assessments at home, and what access to technology and the internet is available
- The intervention content, format and delivery for NHS contexts
- The commissioning of screening and intervention (clinician/commissioner specific)
- The functionality/appropriateness of proposed tests, pathways and outcomes
- Usual care for people with cognitive problems

**Example questions**

1. Using the preliminary logic model (presented to participants in paper format or on a slide) of the proposed cognitive screening and management pathway, can you make any changes to it, based on how you would like the pathway to work?
2. What do you see are the main barriers and facilitators to the delivery of this pathway? What can be done about the barriers?
3. What are your comments about the specific contents of the intervention? What’s missing? What’s not needed?

**Specific questions**

1. Feasibility and acceptability of completing cognitive assessments at home

- When do they receive their appointment letters? (to determine how far in advance letters are sent)
- Have they experienced any appointment cancellations? How many? At what point (e.g., 2 months) before the appointment was scheduled to take place
- To get a sense of where they are at in relation to use of technology, including what they are comfortable doing online
- What kind of things do they do online?
- How do they feel about an online screening platform?

1. Outcomes (avoid using this word to begin with)

- What are the things that matter to pwMS?
- What are the things that are important to you now, and in the future?
- What are the things about your cognitive/general life that you would like to do something about? (this needs to be realistic)
- Are the following outcomes acceptable?
  - Impact of cognitive problems
  - Cognitive impairment
  - Quality of life
  - Mood
  - Function
  - Self-efficacy
  - Resource-use questionnaire
  - Work-related issues
  - Improved DMT adherence
- What outcomes are we not tapping?
- Usual care related to cognitive assessment and intervention
  - If participants have experiences of any cognitive assessments – what they thought of it, or what they would think of receiving it
- Their needs and preferences in terms of location, person administering, modality (online, telephone, face-to-face), and length of tests
- Current screening tools will be presented as a video to participants and they will be asked if they would feel these were appropriate. These tools will include the Symbol Digit Modalities Test (SDMT), the Paced Auditory Serial Addition Test (PASAT) and the California Verbal Learning Test (CVLT).
- Who do you feel comfortable with, in terms of:

1. The person giving you’re the results of the screening
2. The person delivering the cognitive rehabilitation programme?

- MS nurse, occupational therapist?

**Prompts**

Probing questions (Rubin and Rubin, 1995) will be used to transition the conversation from general to specific. That is:

- Requests for extension- e.g. ‘Can you tell me more about....?’, ‘Is there anything else?’, ‘What happened then?’
- Encouraging/prompting questions- e.g. ‘uh huh?’, ‘Yes?’, ‘Please go on’.
- Example questions- e.g. ‘Can you give me an example of?’
- Follow-up questions- e.g. ‘What do you mean by...?’, ‘Would you talk a bit more about...?’

**Coding Scheme**

| Name |
| --- |
| 1.1 Cognitive problems in MS |
| 1.2.1 Identification of cognitive problems |
| 1.2.2 Referral to specialist services for assessment-management of cognitive problems |
| 1.2.3 Management of cognitive problems |
| 1.3 Perceptions of neuropsychological rehabilitation |
| 1.4 Other |
| 2.1.1 Understanding of the rationale purpose of the screening & management pathway |
| 2.1.2 Perceptions of whether the pathway is feasible to deliver within current NHS services-pathways |
| 2.1.3 Perceptions-identification of unmet needs – cognitive screening |
| 2.1.4 Perceptions-identification of unmet needs – referral to specialist services |
| 2.1.5 Perceptions-identification of unmet needs – management of cognitive problems |
| 2.2 Other |
| 3.1.1 Reflections or perceptions of time for completion of screening tests (availability and acceptability) |
| 3.1.2 Reflections or perceptions of time for neuropsychological rehabilitation (availability and acceptability) |
| 3.1.3 Reflections or perceptions of who administers screening test |
| 3.1.4 Reflections or perceptions of who feeds back results of screening test |
| 3.1.5 Reflections or perceptions of who delivers the neuropsychological rehabilitation |
| 3.1.6 Perceptions or reflections of perceived involvement of HCPs in cognitive screening |
| 3.1.7 Perceptions or reflections of perceived involvement of HCPs in referrals |
| 3.1.8 Perceptions or reflections of perceived involvement of HCPs in rehabilitation-management |
| 3.1.9 Perceptions or reflections of perceived involvement of administrative and ICT teams in screening |
| 3.1.10 Perceived involvement of MS charities, commissioners, volunteers, PPI |
| 3.2.1 Content of tests - Perceptions of cognitive screening tests – objective and subjective |
| 3.2.2 Content of tests - Perceptions of self-report measures (not cognition), including proxy measures, including relationship between cognition and other factors (mood, fatigue) |
| 3.2.3 Mode of delivery - Perceptions of screening test formats-modalities – i.e., online, face-to-face, self-administered vs administered by HCP, support vs no support, PC-tablet-mobile phones |
| 3.2.4 Location of testing - Perceptions of screening test completion in clinic – acceptability and feasibility |
| 3.2.5 Location of testing - Perceptions of screening test completion at home – acceptability and feasibility |
| 3.2.6. Facilities - Perceptions of infrastructure requirements for completion of screening tests at home |
| 3.2.7 Facilities - Perceptions of infrastructure requirements for completion of screening tests in clinic |
| 3.3.1 Perceptions of content of the rehabilitation |
| 3.3.2 Perceptions of the acceptability and feasibility of rehabilitation for mild-moderate cognitive problems |
| 3.3.3 Perceptions of the format of delivery – face-to-face, telephone, videoconference, online, group-based, individual |
| 3.3.4 Perceptions of the duration and frequency of sessions |
| 3.3.5 Perceptions of location of rehabilitation – e.g., hospital, community |
| 3.4 Other |
| 4.1 Link - Perceptions of how to send link to patients – including appointment letters, reminder letters, text messages |
| 4.2 Link - Perceptions of how to send screening results to clinicians |
| 4.3 Results of screening - Perceptions of the content of screening results |
| 4.4 Results of screening - Perceptions of accessibility (including understanding), specificity, sensitivity of results to facilitate discussions |
| 4.5 Referral pathway – Perceptions of the appropriateness and acceptability of triaging pwMS (according to low, mild, moderate, severe cognitive problems) |
| 4.6 Referral pathway – Perceived unintended consequences e.g., impact on current services (better identification more patients requiring support and impact on resources). For both MS clinics and community services |
| 4.7 Perceived role of ‘change agents’ such as NICE guidelines, clinical and commissioning toolkits |
| 4.8 Other |
| 5.1.1 Knowledge, beliefs in relation to cognitive screening and management in MS |
| 5.1.2 Perceived understanding of value-benefits of the screening and management pathway |
| 5.1.3 Other potential barriers-enablers to implementation of the pathway – e.g., motivation, perceptions of role individual plays in pathway |
| 5.2.1 Commissioning of services |
| 5.2.2 Culture – e.g., perceived importance of identifying and managing cognitive problems |
| 5.2.3 How services are currently operating – e.g., whether cognitive screening is already being implemented, MDT |
| 5.2.4 Infrastructure-availability of resources |
| 5.3 Other |
| 6.1 Outcome measures (trial) Relationship between cognition and other factors such as mood, fatigue |
| 6.2 Outcome measures (trial) – outcomes that are missing |
| 6.3 Pathway – perceived benefits of pathway on identification and management of cognitive problems in pwMS |
| 6.4 Pathway – Perceived benefits of pathway on utilisation of NHS resources |
| 6.5 Pathway – potential unintended outcomes of pathway on services |
| 6.6 Other |
